# Darnel

**Published:** 1881-10

**Authors:** A. Veitch


### DARNEL.

There seems to be a difference of opinion as to whether the term Darnel is synonymous with rye grass and applied to all the species of Lolium, or whether it should be confined to L. temulentum. Both popular names are applied to all the species by some writers, whilst by others to temulentum only. Hooker uses both indiscriminately, and Gray, Flint, Wood and Chapman follow suit. Mr. P. Henderson in his "Handbook of Plants," in some unaccountable way, says that Darnel is a common name for the Lolium, a genus of noxious grasses introduced from Europe. This cannot be said of L. perenne and Italicum as Mr. H. very well knows, and it would have been more in accordance with popular belief had he said that it is a common name for L. temulentum, a noxious species introduced from Europe. Loudon and Paxton make use of the word in this restricted sense, and in Chambers' Encyclopædia rye grass and Darnel are treated of under separate heads, which is eminently proper. The term is supposed to be derived from the old Saxon word *derian*, to injure, and in one old dictionary it is defined as "The Weed Cockle," which is explicable only on the supposition that from time immemorial it has been regarded with disfavor and classed with the corn cockle (Agrostema Githago), which, when springing up in grain fields, entails both trouble and expense.

This difference of opinion and statement seem to spring from the too-free use of such popular names as have been conferred upon particular plants for obvious or imaginary reasons. And as these reasons become obscured, or are but dimly understood, they are apt to be applied to plants having no connection with those first understood by them.

The popular belief that Darnel is possessed of noxious and poisonous qualities has been much shaken by recent researches on the continent of Europe, as "the effects which have been ascribed to it are now regarded as proceeding from grain injuriously affected in some way by bad weather."—*A. Veitch, in Gardener's Monthly.*

